# Foreground, midground, and restoration: EEG and preference responses to depth-controlled rural scenes

**DOI:** 10.3389/fpsyg.2026.1891123

**Published:** 2026-07-27

**Authors:** Xing Xiong, Shanrui Yang, Xinyi Jiang, Qinghai Zhang

**Affiliations:** Department of Landscape Architecture, Nanjing Agricultural University, Nanjing, China

**Keywords:** depth estimation, EEG, flux, landscape preference, psychological restoration, rural landscape, stress recovery

## Abstract

**Introduction:**

Exposure to natural and rural landscapes has been associated with stress reduction and psychological restoration. However, conventional photograph-based experiments often provide limited control over individual landscape elements and their positions within the visual field. This study examined whether depth-controlled, AI-generated rural landscape stimuli could support stress recovery and clarify how foreground and midground elements relate to landscape preference and EEG-based responses.

**Methods:**

Depth estimation, layer masking, and FLUX-based image editing were used to construct 27 controlled rural landscape stimuli. Selected foreground and midground elements were modified while the background context was kept relatively stable. Thirty participants completed a stress-induction task followed by rural landscape viewing and a duration-matched blank-screen control condition. Systolic blood pressure, diastolic blood pressure, heart rate, EEG beta/alpha (β/α) stress index, and POMS mood scores were measured at baseline, after stress induction, and after recovery. Landscape preference ratings and image-level EEG responses were analyzed for the rural landscape stimuli.

**Results:**

Compared with blank-screen viewing, rural landscape viewing produced significantly greater recovery in all seven stage-level outcomes after Holm correction. Rural-minus-blank recovery differences were −4.45 mmHg for systolic blood pressure, −3.93 mmHg for diastolic blood pressure, −5.85 bpm for heart rate, and −0.10 for the EEG β/α stress index. Within the rural landscape stimuli, foreground water was associated with higher preference ratings (estimate = 0.500, SE = 0.084, *p* < 0.001), whereas foreground road was associated with lower preference (estimate = −0.354, SE = 0.084, *p* < 0.001). EEG-based image-level associations were weaker. Foreground water and forest-based context were associated with lower β/α values in exploratory models, but the matched water-versus-road EEG contrast was not significant after correction.

**Discussion:**

Validated AI-generated rural images can serve as controlled stimuli in environmental psychology research. The findings suggest that rural landscape viewing may promote stronger physiological, EEG-based, and affective recovery than passive blank-screen viewing, while visual preference and EEG-based restoration may follow partly distinct depth-layered pathways. Given the exploratory design, the depth-layer findings should be interpreted cautiously and require confirmation in more systematically balanced stimulus sets.

## 1 Introduction

Stress recovery is a central topic in environmental psychology because everyday environments can either intensify or alleviate psychological and physiological strain. Restorative environment research has shown that natural environments can support affective, attentional, physiological, and neurophysiological recovery, as explained by Stress Recovery Theory and Attention Restoration Theory (Ulrich, 1984; Ulrich et al., 1991; Kaplan and Kaplan, 1989; Kaplan, 1995; Bratman et al., 2019; Frumkin et al., 2017; Markevych et al., 2017). This evidence has been supported by studies using photographs, simulated nature, and virtual reality environments, with outcomes including mood, perceived restoration, cardiovascular responses, skin conductance, and EEG activity (Hartig et al., 2003; Berto, 2005; Berman et al., 2008; Bowler et al., 2010; Hartig et al., 2014; Twohig-Bennett and Jones, 2018; Browning et al., 2020; Song et al., 2022; Chen et al., 2025; Bratman et al., 2015; Rhee et al., 2023; White et al., 2019). University-aged young adults are a relevant group for such research because they often face academic, employment-related, and social pressures (Auerbach et al., 2016; Ribeiro et al., 2018). However, much of this work still focuses on broad environmental categories, such as natural versus urban scenes, or on the general presence of natural elements. Less attention has been given to where specific landscape components appear within the visual field.

Rural landscapes are a meaningful but underexplored context for restorative environment research. Unlike many urban green spaces or single-category natural scenes, rural scenes often combine water bodies, forests, agricultural fields, roads, buildings, and settlement edges within the same visual field. Previous studies have suggested that rural natural perception, place attachment, vegetation, blue–green elements, and landscape coherence may contribute to restorative experience, preference, and emotional responses (Velarde et al., 2007; Jiang et al., 2014; Li and Sullivan, 2016; Tang et al., 2024; Li and Yan, 2026). However, rural landscapes should not be treated simply as less urbanized natural environments. Their restorative potential may depend on how ecological, agricultural, and residential elements are visually organized. Existing restorative landscape studies have mainly compared broad environmental categories or the presence of specific elements, such as water, vegetation, buildings, or roads (Joye and van den Berg, 2011; Lindal and Hartig, 2015). Recent research has further shown that visual composition and vegetation proportion can be associated with attention and attractiveness ratings, although neural responses may be weaker or more complex (Latifzadeh et al., 2026). Unlike Latifzadeh et al. (2026), which examined urban green-space composition and affective responses, this study uses depth-controlled AI-generated rural scenes to examine stress recovery and exploratory foreground–midground associations. These findings suggest that composition matters, but most studies still describe composition through element presence, semantic class, or image proportion. Less attention has been given to the depth position of landscape elements. A water body in the foreground may affect texture, brightness, accessibility, and visual preference, whereas vegetation, fields, or buildings in the midground may contribute more to spatial coherence, enclosure, extent, and the sense of being away. Therefore, a critical unresolved question is not only whether a rural scene contains restorative elements, but how these elements relate to responses when they appear in different visual depth layers.

Recent work has increasingly used controlled images, virtual environments, and computationally generated scenes to examine environmental perception and affective responses. Latifzadeh et al. (2026) is particularly relevant because it used image-based and computational approaches to evaluate affective responses to urban green spaces. It shows the growing value of scalable and controlled visual stimuli for landscape perception research. However, the present study differs from that work in three main respects. First, it focuses on rural scenes in which ecological, agricultural, and settlement elements co-occur, rather than on urban green spaces. Second, it embeds controlled stimuli in a stress-induction and recovery paradigm, rather than focusing only on affective evaluation. Third, it uses depth-estimation-guided editing to examine exploratory foreground, midground, and background-context associations. Therefore, this study does not claim that AI-generated landscape evaluation is entirely new; rather, it extends related work by linking controlled rural scene construction with physiological, EEG-based, and preference responses from a visual-depth perspective.

Studying depth-layered landscape responses requires visual stimuli that are both controlled and credible. Real photographs and field environments offer ecological validity, but they often vary in lighting, viewpoint, season, background composition, and co-occurring elements, which can make it difficult to relate psychological or physiological responses to a specific element or its visual position (Hartig et al., 2014; Holleman et al., 2020; Browning et al., 2020). Manual image editing can improve control, but it may introduce unnatural boundaries, inconsistent illumination, or visible artifacts. AI-assisted image generation and editing offer a possible way to construct more controlled visual stimuli, especially when selected foreground or midground elements need to be modified while other scene features remain comparable (Rombach et al., 2022). However, visual plausibility alone is not sufficient for experimental use. Studies on nature-image validation and simulated nature environments have shown that stimulus properties and presentation medium can influence psychological responses (Browning et al., 2020; Menser et al., 2021). Recent AI-stimulus validation studies also emphasize the need to check key properties such as realism, nameability, familiarity, valence, and arousal patterns (Campbell et al., 2026; Chiu et al., 2026). For environmental psychology, this means that AI-generated rural scenes should be evaluated for realism, semantic recognizability, spatial coherence, depth-layer clarity, and visible artifacts before restoration-related responses are interpreted.

The present study examined whether validated rural landscape stimuli with controlled visual depth can clarify how foreground and midground elements relate to stress recovery, EEG stress indicators, psychological restoration, and landscape preference. Three research questions were addressed: (1) whether the final rural images were realistic and coherent enough to serve as experimental stimuli; (2) whether rural landscape viewing was associated with stronger physiological, EEG, and affective recovery than passive blank-screen viewing; and (3) whether selected foreground, midground, and background context descriptors were associated with differences in preference and EEG β/α responses. We expected that rural landscape viewing would support stronger overall recovery than passive viewing, while depth related associations would differ between preference and EEG responses. Because the stimulus set represented selected rural scene configurations rather than a complete factorial design, the depth related analyses were treated as exploratory. The contribution of this study lies in connecting restorative environment research, controlled stimulus validation, and rural landscape perception from a visual depth perspective.

## 2 Materials and methods

### 2.1 Study design

**Figure 1:**
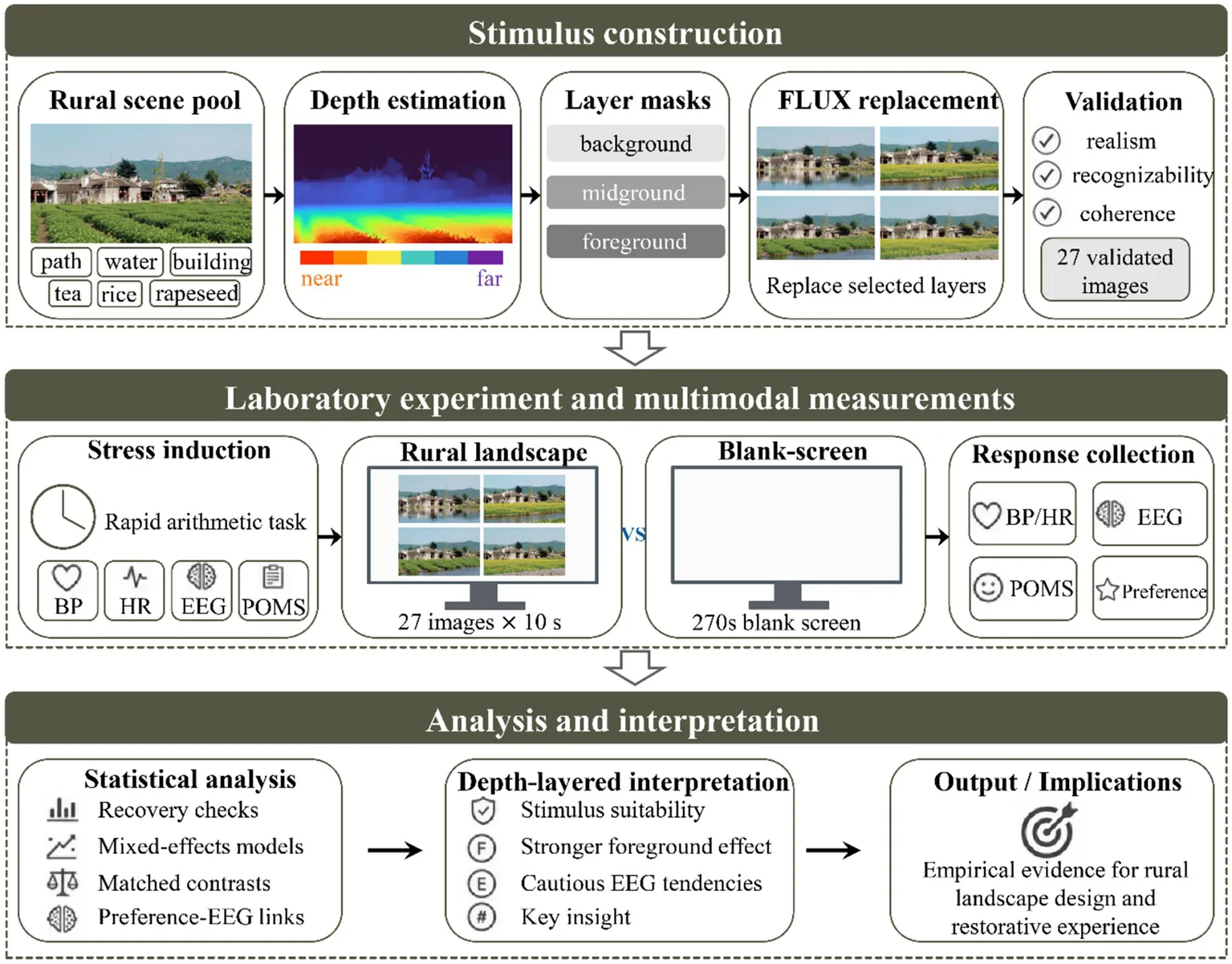
Framework of experiment. The workflow summarizes the construction of 27 depth-controlled rural landscape stimuli, the laboratory stress-recovery experiment with a duration-matched blank-screen condition, and the subsequent analyses. The analyses included stimulus validation, overall recovery comparisons, and exploratory image-level associations between depth-layer descriptors, preference, and EEG β/α responses.

This study used a laboratory-based visual exposure design. The aim was to test whether depth-controlled AI-generated rural landscape images could support stress recovery and landscape preference. The experiment comprised three stages: stimulus construction and validation, stress induction, and recovery. The recovery phase included two conditions: a rural landscape viewing condition and a blank-screen control condition. In the rural landscape condition, participants viewed the 27 final AI-generated images in random order. In the blank-screen condition, they viewed a blank screen for the same total duration. We measured physiological indicators, the EEG-derived stress index, and POMS mood scores at three time points: baseline, immediately after stress induction, and immediately after the recovery phase in each condition. Landscape preference ratings were collected only for the rural landscape stimuli. The analysis was organized at three levels. First, stimulus validation assessed whether the final AI-generated images were sufficiently realistic, semantically recognizable, spatially coherent, and free from dominant visible artifacts. Second, stage-level analyses compared stress induction and recovery between the rural landscape and blank-screen conditions using physiological indicators, EEG β/α stress index, and POMS mood scores. These analyses tested overall recovery during the full viewing period. Third, image-level analyses within the rural landscape condition examined whether preference ratings and EEG β/α values were associated with foreground type, midground type, and background context. Cardiovascular and POMS outcomes were measured only at the stage level and were not used to infer element-specific foreground or midground effects (Figure 1).

### 2.2 Participants

Thirty young adults participated in the experiment (16 males and 14 females; age: 24.23 ± 2.30 years, range 20–27 years). Participants were recruited from Nanjing Agricultural University and related design and environmental disciplines. The sample included students from landscape architecture, environmental design, architecture, urban and rural planning, and horticulture. All participants reported being healthy at the time of the experiment. Inclusion criteria were adult status, normal or corrected-to-normal vision, no self-reported history of neurological or severe psychological disorders, and provision of written informed consent. Participants were asked to avoid alcohol, smoking, and staying up late before the experiment. The study protocol was approved by the local ethics committee of the College of Horticulture, Nanjing Agricultural University (Table 1).

**Table 1:** Participant characteristics.

Characteristic	Category	Value
Sample size	Total participants	30
Age	Years	24.23 ± 2.30; range 20–27
Gender	Male	16 (53.3%)
Gender	Female	14 (46.7%)
Academic background	Landscape architecture	22 (73.3%)
Academic background	Environmental design	3 (10.0%)
Academic background	Architecture	2 (6.7%)
Academic background	Urban and rural planning	2 (6.7%)
Academic background	Horticulture	1 (3.3%)
Rural experience	Occasional rural visits	12 (40.0%)
Rural experience	Rural residence experience	10 (33.3%)
Rural experience	Frequent rural visits	5 (16.7%)
Rural experience	No special rural experience	3 (10.0%)
Health status	Healthy	30 (100.0%)

Values are *n* (%) unless otherwise indicated. Age is reported as mean ± SD.

### 2.3 Construction and validation of AI-generated rural landscape stimuli

Visual stimuli were constructed from rural scene photographs matched for viewpoint, background structure, and viewing distance. The image-editing procedure was designed to control changes in foreground and midground elements while keeping the background stable. Target landscape elements included water, road, rice field, tea field, rapeseed field, and flower field. Building- and forest-dominated base scenes represented contrasting rural spatial contexts. The final stimulus set comprised exclusively AI-generated images; no real photographs were included in the formal experiment. This design avoided mixing real and AI-generated images within the main experiment and allowed the analysis to focus on response differences associated with landscape elements and visual depth layers rather than differences attributable to image source.

**Figure 2:**
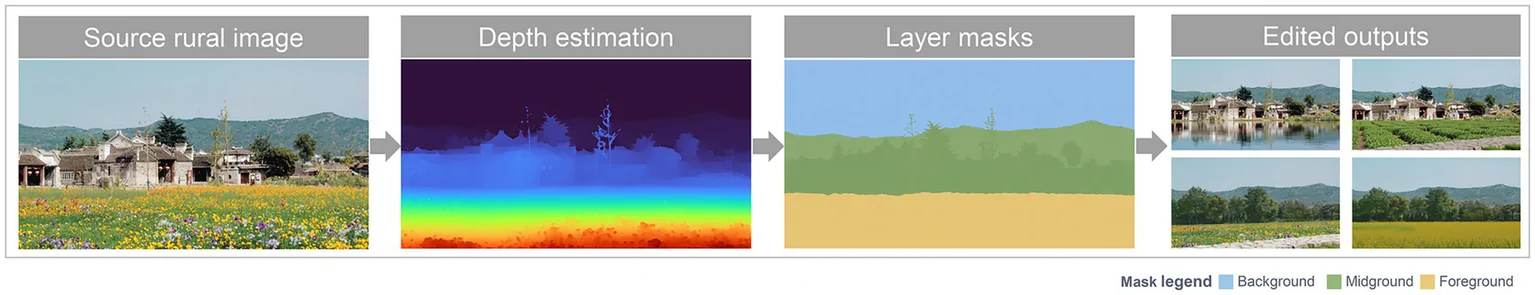
Construction of depth-controlled AI-generated rural landscape stimuli.

For each source image, a relative depth map was generated and used to define foreground, midground, and background layers. Layer masks were then prepared to restrict generative editing to the selected depth layer. FLUX-based image replacement was applied to edit only the target region, while the background and non-target layers were kept as stable as possible. The generated candidates were screened to remove outputs with obvious semantic errors, unnatural boundaries, spatial incoherence, or visible AI artifacts. The final experimental set contained 27 stimuli, including images without foreground or midground replacement, images with foreground replacement only, images with midground replacement only, and images with both foreground and midground replacement. The set was designed to represent selected rural scene configurations with comparable viewpoint and background structure, rather than to cover all possible combinations of foreground, midground, and background context. Accordingly, the image-level analyses were interpreted as exploratory comparisons within this controlled stimulus set (Figure 2 and Table 2).

**Table 2:** Stimulus composition matrix of the 27 final AI-generated rural landscape images.

Background context	Foreground condition	Midground condition	*n*
Building-based context	No foreground replacement	No midground replacement	1
Building-based context	No foreground replacement	Rice, tea, flower, or rapeseed field	4
Building-based context	Water	No midground replacement	1
Building-based context	Water	Rice, tea, flower, or rapeseed field	4
Building-based context	Road	Rice, tea, flower, or rapeseed field	4
Forest-based context	Road	Rice, tea, flower, or rapeseed field	4
Forest-based context	Water	Rice, tea, flower, or rapeseed field	4
Forest-based context	No foreground replacement	Rice, tea, flower, or rapeseed field	4
Forest-based context	Water	No midground replacement	1
Total	/	/	27

The table summarizes the final stimulus composition rather than a fully crossed factorial design. Some cells contain only one image, and not all foreground, midground, and background-context combinations were represented. Midground field categories include rice field, tea field, flower field, and rapeseed field. “No replacement” indicates that the corresponding depth layer was retained from the base scene.

All 27 final stimuli were subjected to stimulus validation. The validation did not compare AI-generated images with real photographs. Instead, it verified whether the final stimulus set was sufficiently realistic, semantically recognizable, spatially coherent, and suitable for use in an environmental psychology experiment. Each image was rated for visual realism, AI-artifact visibility, semantic recognizability, depth-layer clarity, spatial coherence, and perceived naturalness. All validation dimensions were rated on a 5-point Likert-type scale. One indicated very low or poor quality and 5 indicated very high or excellent quality. For AI-artifact visibility, 1 indicated no or minimal visible artifacts and 5 indicated very obvious artifacts. AI-artifact visibility was interpreted in the opposite direction from the other validation dimensions, with lower scores indicating better stimulus quality (Table 3).

**Table 3:** Stimulus validation dimensions.

Dimension	Assessment content	Desired direction
Visual realism	Overall plausibility as a rural landscape scene	Higher
AI-artifact visibility	Visible distortions, unnatural boundaries, or generation artifacts	Lower
Semantic recognizability	Clarity of the target landscape elements	Higher
Depth-layer clarity	Distinction among foreground, midground, and background	Higher
Spatial coherence	Consistency of perspective, scale, boundaries, and spatial relations	Higher
Perceived naturalness	Natural and visually acceptable appearance of the whole scene	Higher

Landscape preference was rated after each rural landscape image on a four-point scale: 0 = dislike, 1 = low preference, 2 = moderate preference, and 3 = high preference.

### 2.4 Experimental procedure

The experiment followed a three-stage physiological and psychological measurement procedure. Participants first rested quietly for 3 min. Baseline systolic blood pressure (SBP), diastolic blood pressure (DBP), heart rate (HR), EEG-derived stress index, and POMS scores were then recorded (McNair et al., 1971). In the stress-induction stage, participants completed a 1-min timed mental-arithmetic task consisting of two-digit addition, subtraction, and multiplication questions. The experimenter immediately corrected incorrect answers and instructed participants to continue, thereby inducing both time pressure and evaluative pressure. Physiological and psychological indicators were recorded immediately after the task as post-stressor measurements.

**Figure 3:**
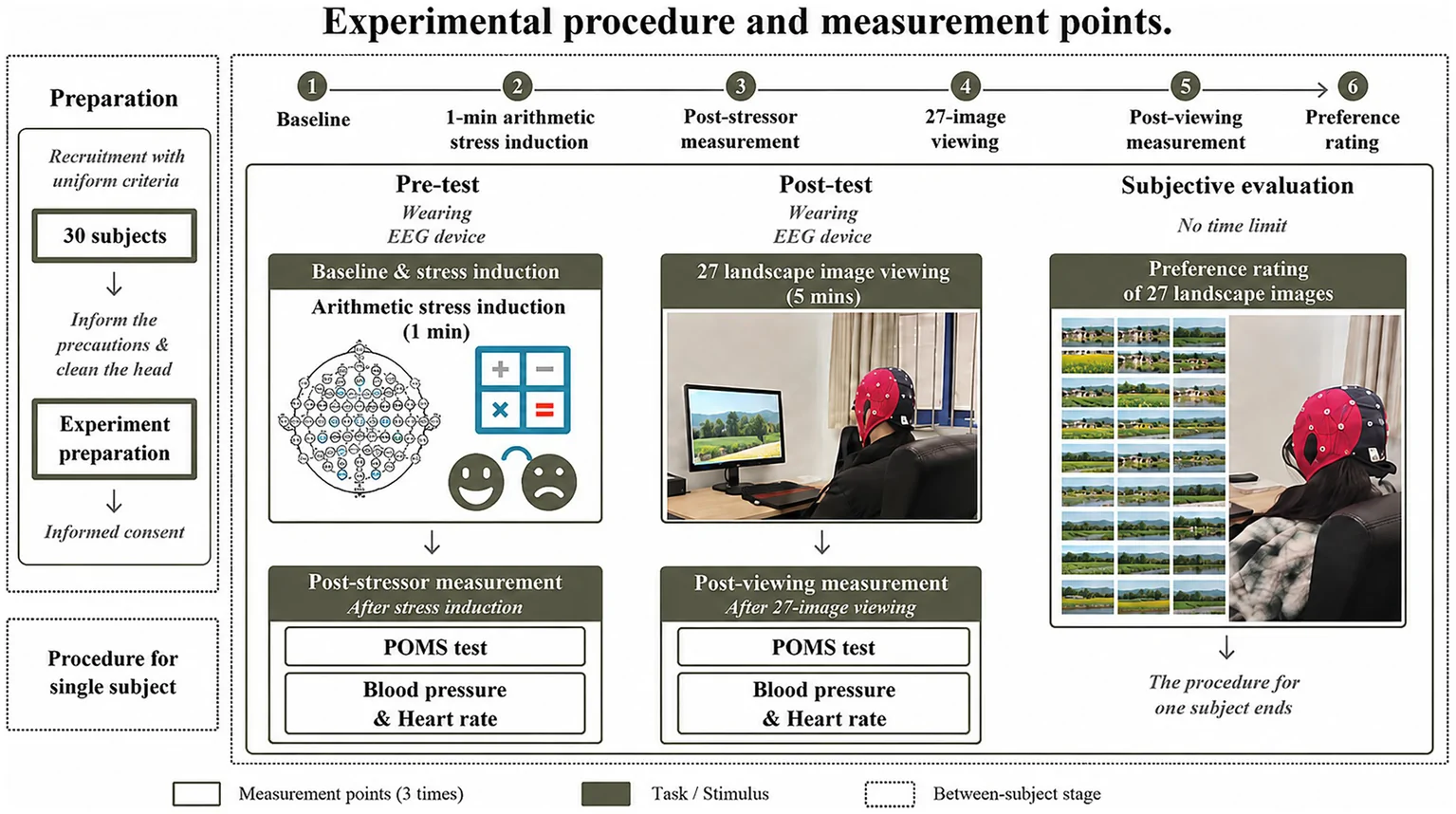
Experimental procedure and measurement points.

During the recovery stage, participants completed both the rural landscape viewing condition and the blank-screen control condition. In the rural landscape viewing condition, the 27 rural landscape stimuli were presented for 10 s each in a randomized order, resulting in a total exposure duration of 270 s. This duration was chosen to allow participants sufficient time to perceive foreground, midground, and background components while keeping the total viewing session short enough to reduce fatigue. The blank-screen condition used the same 270-s duration. This choice is consistent with short-duration visual landscape perception paradigms used in environmental psychology and landscape perception research (Akounach et al., 2025). Viewing distance, screen size, and measurement points were kept consistent across conditions. Physiological, EEG, and psychological measurements were collected immediately after the recovery phase. The stress-induction effect was defined as the change from baseline to post-stressor. The recovery effect was defined as the change from post-stressor to post-viewing or post-blank-screen. Preference ratings were collected for each rural landscape stimulus but were not collected for the blank-screen control condition (Figure 3).

### 2.5 Measurements and data preprocessing

The EEG signals were recorded using a 32-channel eego™ mylab portable EEG system (model ES-303; ANT Neuro, Enschede, Netherlands) and saved with the Eego recording software. Signals were acquired with a waveguard™ cap using Ag/AgCl sintered electrodes arranged according to the extended international 10–20 system (Jasper, 1958). The montage covered frontal, frontocentral, central, centroparietal, temporal, parietal, parieto-occipital, and occipital regions. AFz served as the ground electrode, and CPz was used as the online reference. Raw EEG data were imported into MATLAB and preprocessed using EEGLAB (Delorme and Makeig, 2004). The preprocessing workflow included electrode-coordinate checking, band-pass and notch filtering, artifact rejection, ICA-based removal of ocular and movement-related components, image-locked epoching, and power spectral density estimation. Segments with large artifacts caused by blinks, eye movements, muscle activity, head movement, abrupt voltage shifts, or sustained high-frequency noise were excluded. No channels were excluded after preprocessing; all 32 channels were retained for the whole-scalp EEG β/α analysis. EEG acquisition and preprocessing parameters are summarized in Supplementary Table 1.

Each stimulus image was segmented into a 10 s EEG epoch locked to image onset. The first 1 s was excluded to reduce the influence of stimulus switching and early visual-evoked responses, and the remaining 9 s segment was used for spectral analysis. Mean alpha power was extracted from 8 to 13 Hz, and mean beta power was extracted from 14 to 30 Hz (Klimesch, 1999; Ray and Cole, 1985). The EEG-derived stress index was calculated as the β/α power ratio averaged across retained channels for each epoch. Higher β/α values indicated relatively higher stress-related arousal, whereas lower values indicated stronger relaxation. Because we focused on overall stress-related arousal during rural landscape viewing, the whole-scalp average β/α ratio was used as the primary EEG outcome. Separate region-of-interest analyses were not conducted because the modest sample size and exploratory image-level design did not support stable region-specific inference without adding further multiple-comparison burden.

All 30 participants had valid EEG data for both stage-level and image-level analyses. The image-level EEG dataset included 30 participants and 27 rural landscape stimuli, yielding 810 valid image-level EEG observations. To reduce the influence of individual baseline differences, image-level β/α values were standardized within each participant by converting each value into a z-score based on all valid image epochs from the same participant. Landscape preference ratings, as described in Section 2.3, were collected for each rural landscape stimulus. The preference model used raw 0–3 scores, and model estimates were therefore interpreted on the original preference scale (Table 4).

**Table 4:** Measurement domains and analytical variables.

Outcome measure	Measurement point	Interpretation
SBP, DBP, HR	Baseline, post-stressor, post-recovery	Lower post-recovery values indicate physiological recovery
EEG β/α stress index	Stage-level mean and image-locked epochs	Lower values indicate lower stress-related arousal
POMS negative mood	Baseline, post-stressor, post-recovery	Lower scores indicate affective recovery
POMS positive mood	Baseline, post-stressor, post-recovery	Higher scores indicate affective recovery
POMS TMD-like score	Negative mood minus positive mood	Lower scores indicate better mood state
Landscape preference	Each rural landscape stimulus	0 = dislike, 1 = low preference, 2 = moderate preference, 3 = high preference

### 2.6 Statistical analysis

Analyses were organized at three levels: stimulus validation, stage-level recovery, and image-level responses. Validation scores were summarized at the image level and compared with the midpoint of the 5-point scale; AI-artifact visibility was interpreted in the reverse direction. Stage-level analyses used paired change scores to test stress induction and recovery. Recovery contrasts compared the rural landscape and blank-screen conditions for the seven stage-level outcomes.

Image-level analyses were restricted to the rural landscape condition. Preference ratings and EEG β/α stress index were analyzed using exploratory linear mixed-effects models:


$$Y_{\mathrm{ij}}=\beta_{0}+\beta_{1}\;\text{Foregroun}d_{i}+\beta_{2}\text{Midgroun}d_{i}+\beta_{3}\;\text{Contex}t_{i}+u_{j}+v_{i}+\varepsilon_{\mathrm{ij}}\;$$

where $Y_{\mathrm{ij}}$ denotes the response of participant j to image i , $u_{j}$ is the participant-level random intercept, and $v_{i}$ is the image-level random intercept. The preference model used raw 0–3 scores, whereas the EEG model used within-participant standardized β/α values. Reference levels were building-based context, no foreground replacement, and flower-field midground. Because the stimulus set was not fully crossed, model estimates were treated as exploratory and supplemented with matched contrasts where possible.

Holm correction was applied within predefined outcome families. For stress-induction and stage-level recovery analyses, the correction family included seven outcomes: SBP, DBP, HR, EEG β/α stress index, POMS negative mood, POMS positive mood, and POMS TMD-like score. For image-level matched contrasts, Holm correction was applied separately within the preference contrast family and the EEG β/α contrast family. Uncorrected *p*-values are reported only when explicitly indicated. Stage-level results are reported with mean differences, 95% confidence intervals, and Cohen’s dz. Image-level models report raw estimates and approximate standardized effects where appropriate. No formal *a priori* power analysis was registered before data collection. To clarify the inferential limits of the sample, we conducted sensitivity analyses. With *N* = 30 participants, the stage-level paired comparisons had 80% power to detect effects of approximately Cohen’s dz. = 0.53 at *α* = 0.05. Therefore, the stage-level recovery analyses were better suited to detecting medium-to-large within-subject effects. The image-level mixed models included 810 subject-image observations but only 27 unique stimuli; therefore, small EEG associations and element-specific estimates should be interpreted cautiously, especially because the stimulus matrix was not fully crossed.

## 3 Results

### 3.1 Stimulus validation and stress manipulation checks

**Figure 4:**
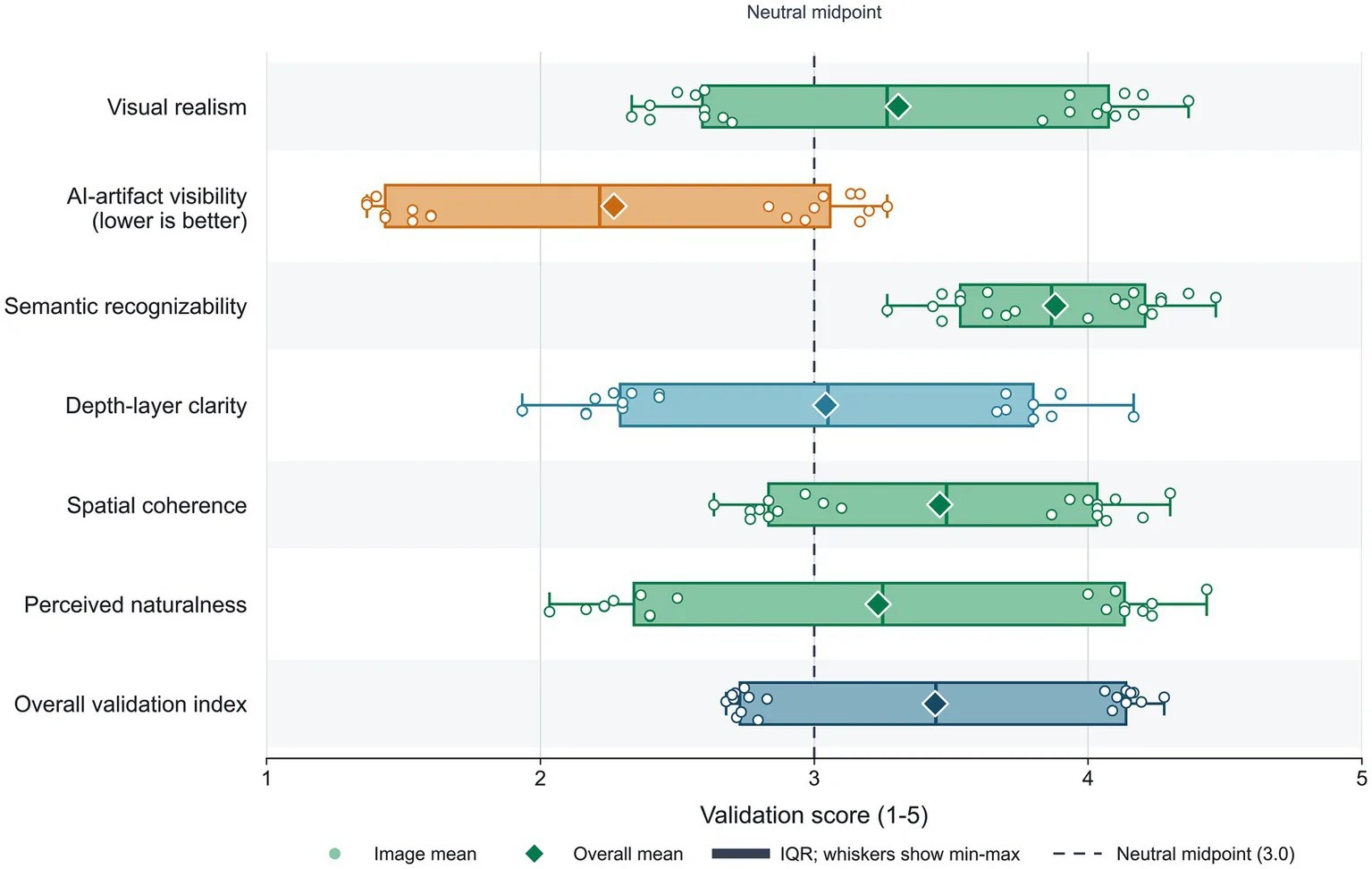
Image-level validation profile of the final AI-generated rural landscape stimuli. Circles show image means, boxes show IQRs, whiskers show min–max ranges, and diamonds show overall means. The dashed line marks the neutral midpoint (3.0); lower AI-artifact scores indicate better quality.

The validation analysis indicated that the 27 final AI-generated rural landscape stimuli were generally suitable for controlled visual exposure, although support varied across dimensions. Across all validation ratings, visual realism was above the neutral midpoint (*M* = 3.31, SD = 1.03), semantic recognizability was relatively high (*M* = 3.88, SD = 0.74), spatial coherence was positive (*M* = 3.46, SD = 0.91), and perceived naturalness was also above the neutral midpoint (*M* = 3.23, SD = 1.17). AI-artifact visibility was below the neutral midpoint (*M* = 2.27, SD = 1.04), indicating that visible generation artifacts were not dominant in the final stimulus set. The overall validation index was above the neutral midpoint (*M* = 3.44, SD = 0.77). Depth-layer clarity, however, was close to the neutral midpoint (*M* = 3.04, SD = 1.08), suggesting that the foreground and midground manipulations were recognizable but not equally clear across all images. The stimulus set was therefore considered suitable for the present experiment, while the interpretation of depth-layer associations remained cautious (Figure 4).

The stress-induction manipulation was effective in both recovery conditions. From baseline to post-stressor, physiological, EEG-based, and POMS indicators changed in the expected direction: SBP, DBP, HR, EEG β/α stress index, POMS negative mood, and POMS TMD-like scores increased, whereas POMS positive mood decreased. After Holm correction across the seven outcomes, the magnitude of stress induction did not differ significantly between the rural landscape and blank-screen conditions. This pattern suggests broadly comparable pre-recovery stress levels across the two conditions, although the absence of significant differences should not be interpreted as formal equivalence.

### 3.2 Overall recovery after rural landscape viewing

**Figure 5:**
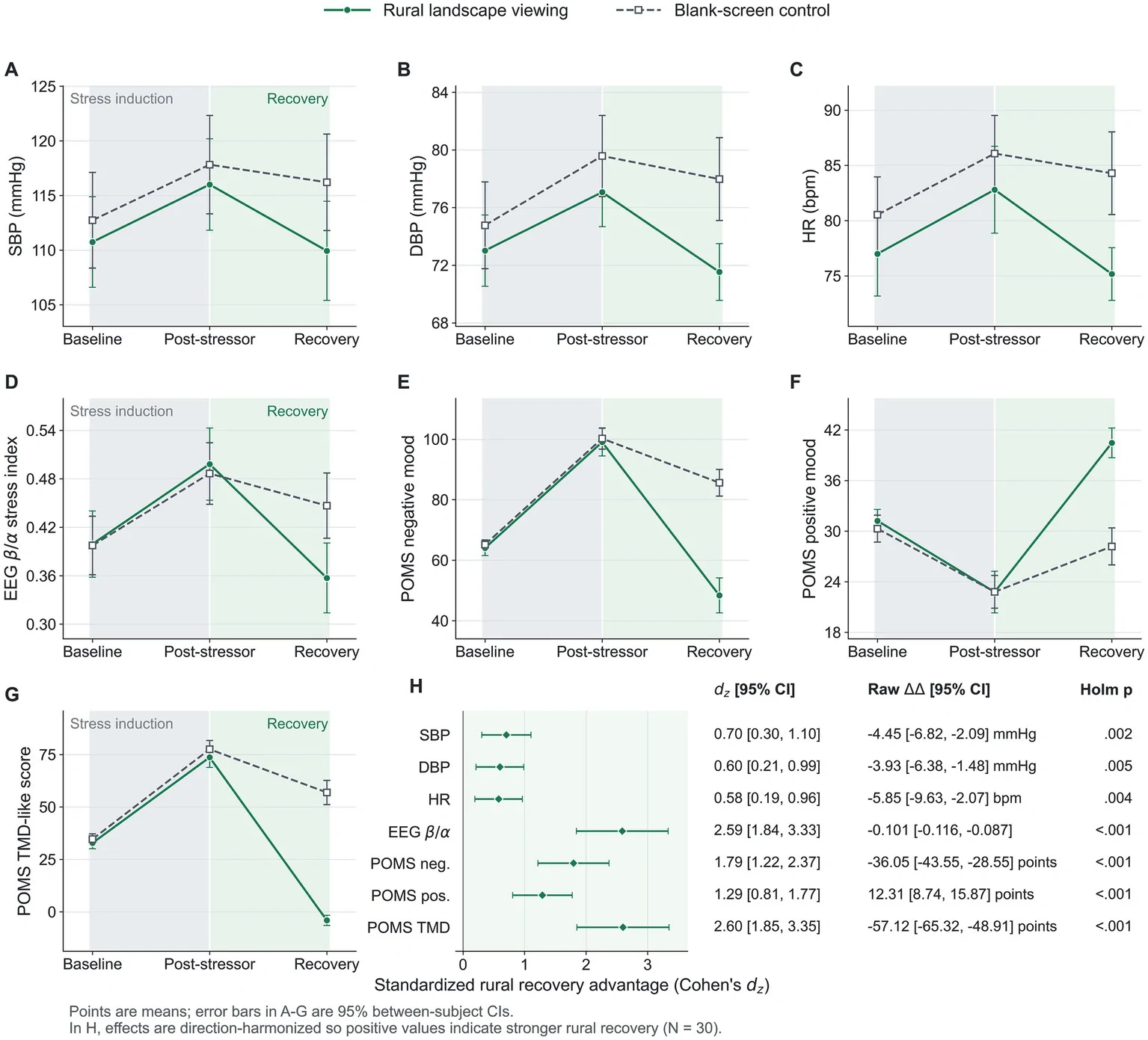
Stage-level trajectories and rural landscape recovery advantages across physiological, EEG-based, and affective outcomes. **(A–G)** Changes in SBP, DBP, HR, EEG β/α stress index, POMS negative mood, POMS positive mood, and POMS TMD-like score across baseline, post-stressor, and recovery phases. **(H)** Overall recovery advantages of rural landscape viewing relative to the blank-screen control, including Cohen’s dz, raw recovery differences, 95% confidence intervals, and Holm-corrected p-values.

This section reports recovery from the post-stressor measurement to the post-recovery measurement. The analyses are based on stage-level outcomes and reflect aggregate change during the full viewing period. They do not isolate responses to specific foreground elements, midground elements, or individual images; those image level analyses are reported in Section 3.3 (Figure 5).

Both conditions showed recovery after stress induction, but the magnitude of recovery was greater after rural landscape viewing than after blank-screen viewing. Paired comparisons of recovery magnitude showed significant rural versus blank differences for all seven outcomes after Holm correction (Table 5). Compared with blank-screen viewing, rural landscape viewing produced larger decreases in SBP, DBP, and HR, with rural minus blank differences of −4.45 mmHg, −3.93 mmHg, and −5.85 bpm, respectively. The EEG β/α stress index also showed a larger reduction after rural landscape viewing (difference = −0.10, 95% CI [−0.12, −0.09]). For affective outcomes, rural landscape viewing produced larger reductions in POMS negative mood and POMS TMD-like score, and a larger increase in POMS positive mood.

**Table 5:** Stage level recovery outcomes: rural landscape viewing versus blank screen control.

Indicator	Rural Δ *M* ± SD	Blank *Δ M* ± SD	*Δ* Difference [95% CI]	Cohen’s dz [95% CI]	Holm *p*
SBP (mmHg)	−6.07 ± 5.68	−1.61 ± 3.60	−4.45 [−6.82, −2.09]	−0.70 [−1.10, −0.30]	0.002
DBP (mmHg)	−5.53 ± 6.02	−1.60 ± 1.44	−3.93 [−6.38, −1.48]	−0.60 [−0.99, −0.21]	0.005
HR (bpm)	−7.63 ± 9.56	−1.79 ± 2.91	−5.85 [−9.63, −2.07]	−0.58 [−0.96, −0.19]	0.004
EEG β/α stress index	−0.14 ± 0.04	−0.04 ± 0.02	−0.10 [−0.12, −0.09]	−2.59 [−3.33, −1.84]	<0.001
POMS Negative Mood	−50.65 ± 21.26	−14.60 ± 10.14	−36.05 [−43.55, −28.55]	−1.79 [−2.37, −1.22]	<0.001
POMS Positive Mood	+17.69 ± 8.28	+5.39 ± 4.30	+12.31 [8.74, 15.87]	+1.29 [0.81, 1.77]	<0.001
POMS TMD-like	−77.71 ± 15.08	−20.59 ± 16.56	−57.12 [−65.32, −48.91]	−2.60 [−3.35, −1.85]	<0.001

*N* = 30. Δ = post-recovery minus post-stressor. Δ Difference = Rural Δ minus Blank Δ. Negative values indicate larger reductions in the rural condition for SBP, DBP, HR, EEG β/α, POMS Negative Mood, and POMS TMD-like. Positive values indicate a larger increase in the rural condition for POMS Positive Mood. Cohen’s dz is the standardized mean difference for paired comparisons. Holm correction was applied across the seven recovery outcomes.

These findings indicate that rural landscape viewing supported recovery beyond passive viewing. The blank-screen condition served as a passive recovery reference; it was not intended as a comparison with other visual environments such as urban scenes, real photographs, videos, virtual reality, or field settings. Because SBP, DBP, and HR were measured at the phase level, these cardiovascular outcomes should be interpreted as evidence of overall physiological recovery rather than responses to specific depth layer elements. Section 3.3 therefore focuses on image-level EEG and preference outcomes within the rural landscape condition.

### 3.3 Foreground and midground associations with EEG stress index and preference

As reported in Section 3.2, rural landscape viewing as a whole produced stronger physiological, EEG, and affective recovery than passive blank-screen viewing. The following analyses focus on differences among the 27 rural landscape stimuli. Because the stimulus set was not fully crossed across foreground, midground, and background context, these results are interpreted as exploratory image-level associations rather than independent causal effects of specific landscape elements.

Landscape preference showed a clear foreground-related pattern. In the mixed model, foreground water was associated with higher preference on the 0–3 scale (estimate = +0.500, SE = 0.084, *p* < 0.001, approximate standardized effect = 0.49), whereas foreground road was associated with lower preference (estimate = −0.354, SE = 0.084, *p* < 0.001, approximate standardized effect = −0.35). Base scene did not significantly predict preference (forest vs. building: estimate = −0.121, SE = 0.066, *p* = 0.066). Among midground elements, rapeseed field (estimate = −0.233, SE = 0.097, *p* = 0.016) and tea field (estimate = −0.344, SE = 0.097, *p* < 0.001) were associated with lower preference relative to flower field, whereas rice field was not significantly different from flower field.

Matched contrasts supported the foreground preference pattern. When water and road foreground images were compared within matched base scene and midground conditions, water foreground images received higher preference ratings than road foreground images (mean difference = +0.95, *Cohen’s dz* = +0.68, Holm-adjusted *p* < 0.001). The result indicates that, within the present stimulus set, visually salient foreground elements were strongly associated with immediate preference.

The EEG β/α responses showed a weaker and less consistent pattern. In the mixed model, foreground water was associated with a lower EEG β/α stress index (estimate = −0.062, SE = 0.023, *p* = 0.007), whereas foreground road was not significantly different from the no foreground condition (estimate = −0.029, SE = 0.023, *p* = 0.204). Forest base scenes were also associated with lower EEG β/α values than building base scenes (estimate = −0.037, SE = 0.017, *p* = 0.030). However, the matched water versus road contrast for EEG was not significant (mean difference = −0.049, *Cohen’s dz* = −0.11, uncorrected *p* = 0.102, Holm-adjusted *p* = 0.305).

**Figure 6:**
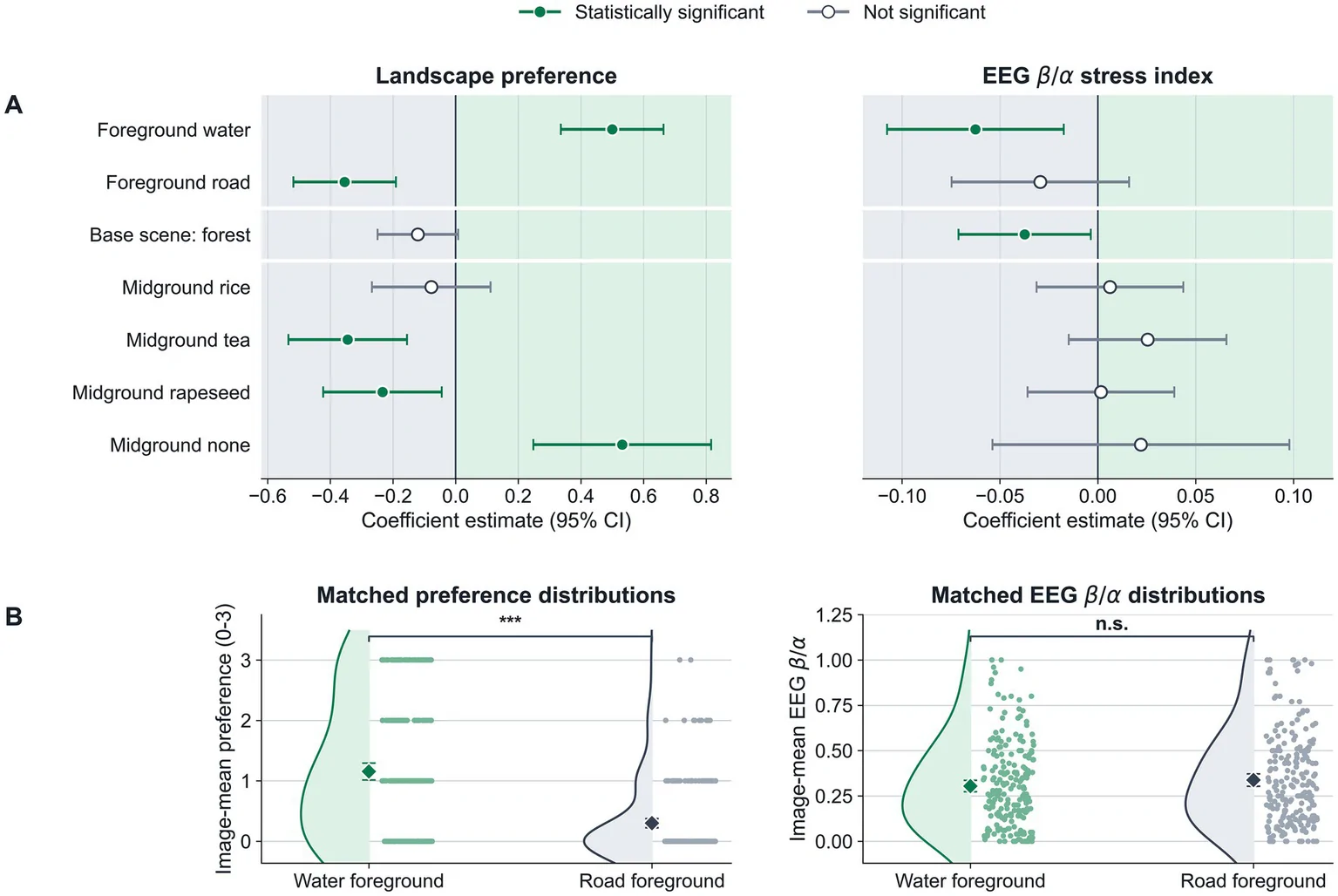
Mixed-model associations and matched water–road distributions for landscape preference and EEG stress responses. Image-level associations between depth-layered landscape elements, landscape preference, and the EEG β/α stress index. **(A)** Fixed-effect estimates and 95% confidence intervals from exploratory mixed-effects models. Reference levels were building base scene, no foreground element, and flower-field midground. Filled green points indicate statistically significant coefficients; open gray points indicate non-significant coefficients. **(B)** Half-raincloud plots comparing water- and road-foreground images across eight matched base-scene-by-midground strata. Density curves show the observed distributions, small points represent participant-by-image observations, diamonds indicate means, and error bars indicate 95% confidence intervals. Preference included 240 observations per foreground condition; EEG included 216 observations per condition after exclusion of invalid EEG records. Brackets report Holm-adjusted matched contrasts (***p < 0.001; n.s. *p* ≥ 0.05). Lower EEG β/α values indicate lower stress-related arousal.

Taken together, the image-level results suggest different response patterns for preference and EEG. Preference was strongly associated with foreground composition, especially the contrast between water and road. EEG β/α responses showed only tentative associations with foreground water and forest-based context. These findings should therefore be interpreted cautiously. They suggest that visually preferred rural images may not necessarily produce the strongest EEG-based relaxation, and that EEG responses may depend on broader scene context rather than foreground elements alone (Figure 6).

### 3.4 Different patterns in preference and EEG recovery

The preceding analyses showed that preference and EEG responses did not follow the same pattern across depth-layered elements. Preference was clearly foreground-driven: foreground water increased preference on the raw 0–3 scale (estimate = 0.500, SE = 0.084, *p* < 0.001; approximate standardized effect = 0.49), whereas foreground road reduced preference (estimate = −0.354, SE = 0.084, *p* < 0.001; approximate standardized effect = −0.35). By contrast, EEG-based responses were weaker. Foreground water and forest-based spatial context were associated with lower EEG β/α stress index values in the exploratory model, but the matched contrasts were not uniformly significant after correction.

The association between preference and EEG-based recovery also depended on the level of analysis. At the image level, higher mean preference was moderately associated with lower mean EEG β/α stress index (Pearson *r* = 0.474, 95% CI [0.115, 0.724], *p* = 0.012; Spearman rho = 0.474, *p* = 0.013). However, this association was weak and non-significant when all subject-image observations were pooled (*r* = 0.039, 95% CI [−0.030, 0.108], *p* = 0.287). Within-participant correlations also varied substantially, with a mean r of 0.052 and a range from −0.443 to 0.371.

**Figure 7:**
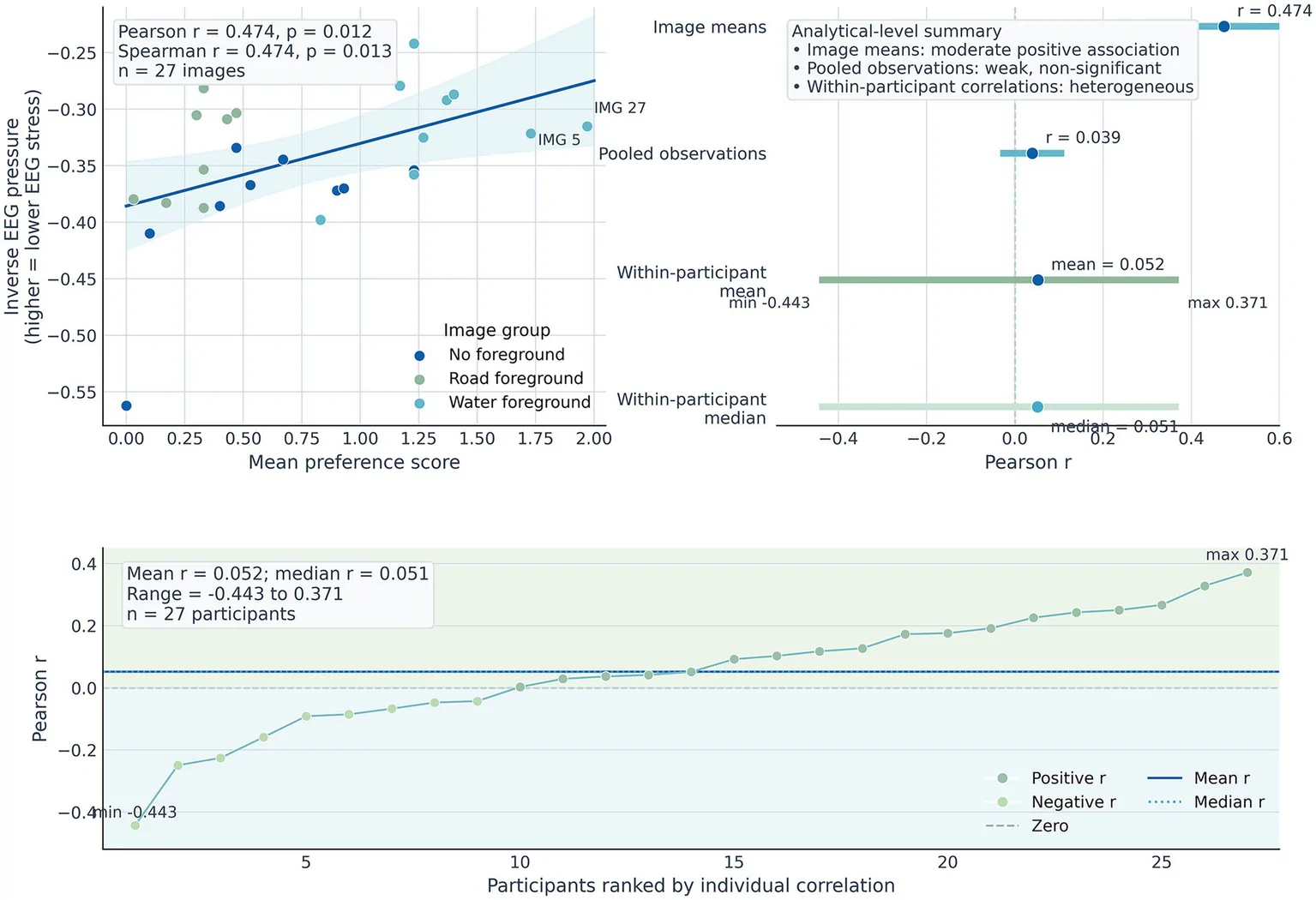
Response-specific relationship between landscape preference and EEG-based restoration across image, observation, and participant levels. Image-level preference was moderately associated with inverse EEG β/α stress index, whereas pooled and within-participant associations were weak. Higher inverse EEG β/α values indicate lower EEG stress.

These findings indicate that the preference–EEG association was present only at the image-mean level. It was not stable when subject-image observations were pooled or when within-participant correlations were examined. Preference should therefore not be treated as a substitute for EEG-based recovery (Figure 7).

These findings suggest that visual preference and EEG-based restoration were partially related but not interchangeable. Foreground elements, especially water and road surfaces, mainly shaped immediate visual appraisal, whereas EEG-based stress responses appeared more sensitive to broader natural context and foreground water but with weaker statistical support. Therefore, the depth-layered effects observed in this study should be interpreted as response-specific: visually salient foreground elements were more strongly associated with preference, while broader natural context may have contributed to EEG-related relaxation.

## 4 Discussion

This study examined whether visual depth layers in controlled rural landscape stimuli were associated with short-term stress recovery and landscape preference. Using depth-estimation-guided image editing, we constructed validated AI-generated rural scenes as controlled visual materials rather than treating AI generation as the main contribution. Three findings emerged. First, the stimuli were generally realistic, recognizable, coherent, and visually natural, although depth-layer clarity was moderate. Second, rural landscape viewing was associated with stronger physiological, EEG-based, and affective recovery than blank-screen passive viewing. Third, image-level associations differed by response measure: foreground water and road were more clearly related to preference, whereas EEG-based responses showed weaker associations with foreground water and forest-based context. These results suggest that restorative responses to rural landscapes are shaped by both visual depth position and the response system being measured.

### 4.1 Controlled AI-generated stimuli in restorative environment research

A central methodological issue in restorative environment research concerns balancing ecological validity and experimental control. Real photographs, videos, and field settings preserve many qualities of actual environments, but they hinder isolation of the effect of a single landscape element or its position in the visual field. Prior reviews of simulated natural environments have shown that photographs, videos, and virtual environments can support mood, stress, and restorative outcomes, but also that methodological choices strongly influence the observed effects (Browning et al., 2021; Spano et al., 2023). In rural scenes, water, farmland, roads, buildings, vegetation, lighting, viewing angle, seasonal condition, and background composition co-vary. As a result, observed differences in stress recovery or preference may reflect multiple uncontrolled visual factors rather than the target element alone.

The present study addressed this issue by combining depth estimation with FLUX-based masked replacement. This approach enabled controlled construction of rural landscape stimuli. Its value lies not in generative AI per se, but in the explicit control of visual layers and editing regions. By defining foreground and midground areas through depth estimation and manual inspection, the procedure enabled manipulation of selected landscape elements while maintaining a relatively stable background. This establishes a clearer link between stimulus construction and experimental interpretation than conventional photo selection or manual editing, especially when the research question concerns the visual position of landscape elements.

Stimulus validation was thus essential. Because all formal stimuli were AI-generated, the aim was not to show that AI images were superior to real photographs, but to verify that the final stimuli were realistic, coherent, and semantically interpretable enough for a controlled visual exposure experiment. This requirement is consistent with recent work showing that AI-generated stimulus sets should be validated for realism, recognizability, familiarity, or emotional quality before being used in behavioral or affective research (Campbell et al., 2026; Chiu et al., 2026). The validation results generally met this requirement: realism, semantic recognizability, spatial coherence, perceived naturalness, and the overall validation index were above the neutral midpoint, while perceived AI-artifact visibility remained low. However, depth-layer clarity was close to the neutral midpoint, suggesting that the visual separation of foreground, midground, and background was not equally strong for all images. This result supports the use of the stimuli but also justifies a cautious interpretation of depth-layer associations.

The blank-screen control condition strengthens the interpretation of these recovery results. Passive recovery did occur during blank-screen viewing. This confirms that part of the post-stressor decrease in arousal reflected natural recovery over time—but only part. Rural landscape viewing produced consistently larger reductions across physiological indicators, EEG β/α stress index, and POMS mood measures. This comparison suggests that controlled rural landscape exposure contributed additional recovery beyond passive rest. At the same time, the blank-screen condition should not be interpreted as a substitute for other visual environments. It controls for time and passive viewing, but it does not determine whether AI-generated rural landscapes are more restorative than urban scenes, indoor scenes, real photographs, videos, or immersive field environments.

### 4.2 Depth position, preference, and EEG recovery

The key theoretical implication of this study is that rural landscape elements may be understood not only by semantic category, but also by visual depth layer and response type. This interpretation is consistent with landscape-perception theories that treat visual quality as a combination of naturalness, coherence, complexity, visual scale, and spatial organization rather than as a simple inventory of objects (Tveit et al., 2006; Ode et al., 2008; Parsons and Daniel, 2002). The preference results suggested a clear foreground-related pattern. Foreground water substantially increased landscape preference. Foreground road reduced it. Near-field elements may exert a strong influence on immediate visual appraisal: they occupy a salient position in the visual field and directly shape perceived texture, brightness, accessibility, and naturalness. In rural landscapes, a water edge or a soft agricultural foreground may provide an attractive visual entry into the scene. A visually dominant road surface, by contrast, may introduce a harder and more artificial impression.

The EEG-based results were less direct. The exploratory model suggested that foreground water and forest-based spatial context were associated with lower EEG stress index values, but conservative matched contrasts did not provide uniformly corrected significant evidence. Rather than demonstrating that a particular element reliably produces stronger neural restoration, the findings suggest a more tentative pattern: EEG-related relaxation may depend on broader spatial coherence, vegetation continuity, and the overall natural context of the scene. Forest-related backgrounds or vegetation-continuous contexts may provide a less visually demanding setting, which could support being away, fascination, and perceived restorative quality (Hartig et al., 1997; Kaplan, 1995; Laumann et al., 2001). This interpretation requires further testing with larger samples and more balanced stimulus sets.

The response-specific pattern is meaningful because preference and EEG-based restoration were not interchangeable. At the image level, preferred stimuli tended to show lower EEG stress index values, indicating that some visual features may jointly support aesthetic liking and relaxation. However, this association disappeared when all subject-image observations were pooled and when within-participant correlations were examined. This result is consistent with prior work showing that environmental preference and restoration are related but not identical constructs (van den Berg et al., 2003; Korpela et al., 2001). Preference may be more strongly shaped by salient visual features, familiarity, personal taste, rural experience, color, and compositional clarity. EEG β/α responses, by contrast, reflect a physiological dimension of arousal and relaxation that may not always align with conscious liking.

**Figure 8:**
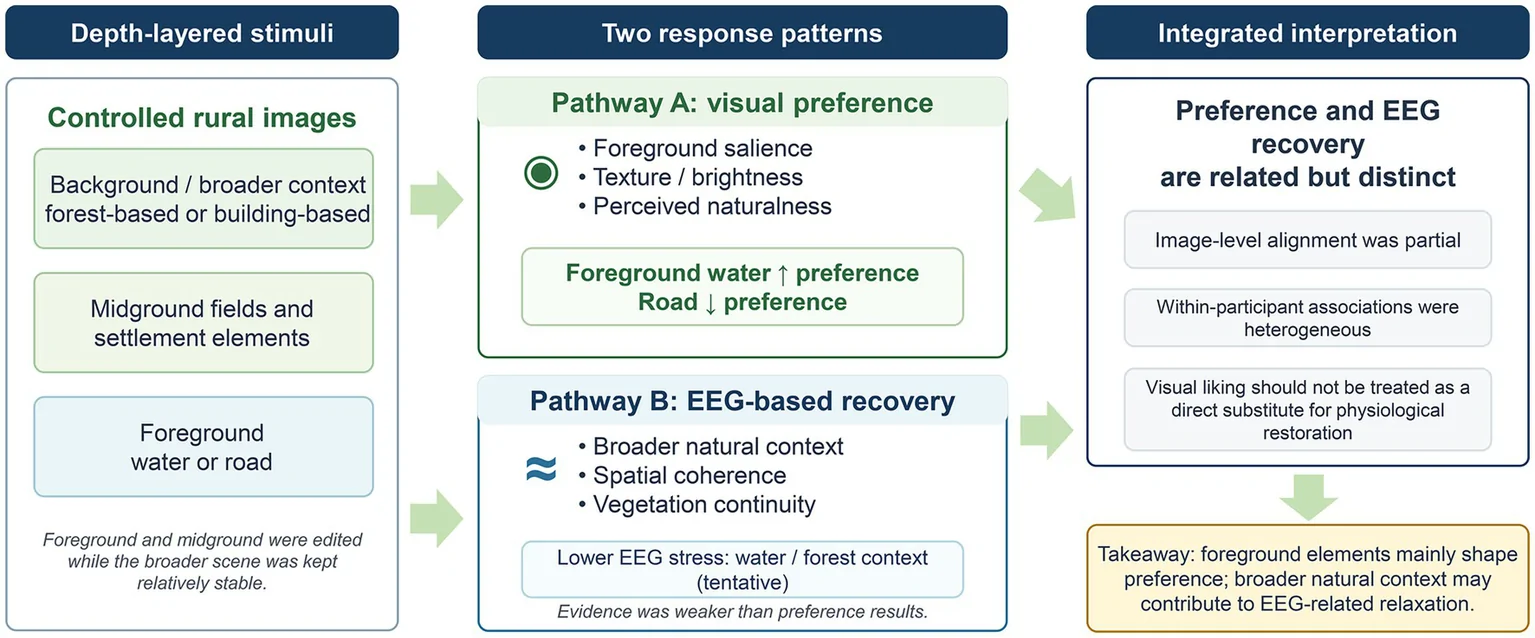
Response-specific pathways of depth-layered rural landscape perception.

These results refine the way restorative rural landscapes can be understood. A rural scene may be visually preferred without necessarily producing the strongest EEG-based recovery for every participant. Conversely, a spatial context that supports lower stress-related arousal may not always be rated as the most attractive. Restorative landscape research should therefore avoid treating preference as a direct substitute for physiological restoration (van den Berg et al., 2003; Herzog et al., 2003). A depth-layered perspective helps explain this complexity: foreground elements may mainly shape immediate visual preference, whereas broader contextual elements may contribute to EEG-related relaxation through spatial coherence, continuity, enclosure, and the sense of being away (Figure 8).

Foreground elements are proposed to mainly influence immediate visual preference through salient texture, color, and perceived naturalness, whereas broader natural context may contribute to EEG-related relaxation through spatial coherence, vegetation continuity, and being away. The partial and scale-dependent association between preference and EEG-based restoration suggests that visual liking and physiological relaxation should be treated as related but distinct response pathways.

### 4.3 Implications and limitations

The findings offer cautious implications for restorative rural landscape design. Foreground composition may shape immediate visual preference. Natural foreground elements, such as water edges and soft agricultural textures, may increase the attractiveness of rural scenes, whereas visually dominant road surfaces or hard artificial materials in the near field may reduce preference and perceived naturalness. This does not suggest that roads should be removed from rural environments, but that their visual dominance, material texture, edge treatment, and integration with vegetation or agricultural fields should be considered when restorative visual experience is a design goal.

The results also suggest that restorative rural landscapes should not be understood only through foreground features. Broader spatial organization, including vegetation continuity, field–forest relationships, midground coherence, and a sense of extent or being away, may also contribute to restorative experience. However, the EEG evidence was exploratory and was not consistently supported by corrected matched contrasts. These design implications should therefore be treated as hypotheses for future testing rather than as direct design prescriptions.

Several limitations should be noted. The sample was mainly composed of young adults from design and environmental disciplines, which may have influenced landscape preference and limits generalizability to broader populations. The blank-screen condition helped distinguish rural landscape viewing from passive recovery, but it did not compare rural scenes with other visual environments. The use of static AI-generated images means that the results reflect controlled visual exposure rather than real rural experience. In addition, the stimulus set was not fully crossed across foreground, midground, and background context, and depth-layer clarity was only moderate. The image-level findings should therefore be interpreted as exploratory associations rather than definitive evidence of independent element effects. Although sensitivity analyses indicated that the study was better powered for stage-level recovery and preference effects than for subtle EEG differences, no *a priori* power analysis was registered. The whole-scalp EEG approach also cannot identify region-specific neural mechanisms. Future studies should use larger and more diverse samples, clearer and more balanced stimulus sets, comparisons with other visual environments, and multimodal measures such as eye tracking, heart-rate variability, skin conductance, or qualitative reports.

## 5 Conclusion

This study used depth-controlled AI-generated rural landscape stimuli to examine stress recovery, visual preference, and EEG-based responses to foreground and midground landscape elements. The validated stimulus set was generally realistic, coherent, and semantically recognizable. Compared with blank-screen viewing, rural landscape viewing produced stronger physiological, EEG-based, and affective recovery after stress induction. Within the rural landscape stimuli, foreground water was associated with higher preference, whereas foreground road was associated with lower preference. EEG-based responses showed weaker and less consistent tendencies related to foreground water and forest-based spatial context. These findings suggest response-specific depth-layered associations: foreground elements were more closely related to immediate visual preference, while broader natural context may have contributed to EEG-related relaxation. Preference and EEG-based restoration were only partially aligned, suggesting that visual liking should not be treated as a direct substitute for physiological restoration. Overall, the study demonstrates that validated AI-generated images can serve as controlled visual stimuli for environmental psychology research, while further validation in balanced, immersive, and real-world settings remains necessary.

## Data Availability

The raw data supporting the conclusions of this article will be made available by the authors, without undue reservation.

## References

[ref1] Akounach M. Lelard T. Mouras H. (2025). Postural correlates of pleasant landscapes visual perception. Front. Psychol. 16:1527691. doi: 10.3389/fpsyg.2025.1527691, 39973947 PMC11837975

[ref2] Auerbach R. P. Alonso J. Axinn W. G. Cuijpers P. Ebert D. D. Green J. G. . (2016). Mental disorders among college students in the World Health Organization world mental health surveys. Psychol. Med. 46, 2955–2970. doi: 10.1017/S0033291716001665, 27484622 PMC5129654

[ref3] Berman M. G. Jonides J. Kaplan S. (2008). The cognitive benefits of interacting with nature. Psychol. Sci. 19, 1207–1212. doi: 10.1111/j.1467-9280.2008.02225.x, 19121124

[ref4] Berto R. (2005). Exposure to restorative environments helps restore attentional capacity. J. Environ. Psychol. 25, 249–259. doi: 10.1016/j.jenvp.2005.07.001

[ref5] Bowler D. E. Buyung-Ali L. M. Knight T. M. Pullin A. S. (2010). A systematic review of evidence for the added benefits to health of exposure to natural environments. BMC Public Health 10:456. doi: 10.1186/1471-2458-10-456, 20684754 PMC2924288

[ref6] Bratman G. N. Anderson C. B. Berman M. G. Cochran B. de Vries S. Flanders J. . (2019). Nature and mental health: an ecosystem service perspective. Sci. Adv. 5:eaax0903. doi: 10.1126/sciadv.aax0903, 31355340 PMC6656547

[ref7] Bratman G. N. Hamilton J. P. Hahn K. S. Daily G. C. Gross J. J. (2015). Nature experience reduces rumination and subgenual prefrontal cortex activation. Proc. Natl. Acad. Sci. 112, 8567–8572. doi: 10.1073/pnas.1510459112, 26124129 PMC4507237

[ref8] Browning M. H. E. M. Mimnaugh K. J. van Riper C. J. Laurent H. K. LaValle S. M. (2020). An actual natural setting improves mood better than its virtual counterpart: a meta-analysis of experimental data. Front. Psychol. 11:2200. doi: 10.3389/fpsyg.2020.02200 PMC7554239 33101104

[ref9] Browning M. H. E. M. Saeidi-Rizi F. McAnirlin O. Yoon H. Pei Y. (2021). The role of methodological choices in the effects of experimental exposure to simulated natural landscapes on human health and cognitive performance: a systematic review. Environ. Behav. 53, 687–731. doi: 10.1177/0013916520906481

[ref10] Campbell G. Nicholls G. Hart R. Allen R. J. von Bastian C. C. Burke M. R. . (2026). Development and validation of an AI-generated real-world object stimuli set. Behav. Res. Methods 58:154. doi: 10.3758/s13428-026-03021-0, 42086798 PMC13144173

[ref11] Chen J. Zhu H. Cheng Y. Yin H. Yi M. Shen D. . (2025). Restorative effects and perception of nature-themed artworks in indoor environments: an empirical study based on VR+EEG. Front. Psychol. 16:1571176. doi: 10.3389/fpsyg.2025.1571176, 40703729 PMC12283990

[ref12] Chiu H. T. Sou H. I. Lam Y. W. Ng C. S. F. Wong S. W. (2026). Beyond traditional stimuli: validating AI-generated images for eliciting negative emotions in affect research. PLoS One 21:e0342434. doi: 10.1371/journal.pone.0342434, 41666168 PMC12890096

[ref13] Delorme A. Makeig S. (2004). EEGLAB: an open source toolbox for analysis of single-trial EEG dynamics including independent component analysis. J. Neurosci. Methods 134, 9–21. doi: 10.1016/j.jneumeth.2003.10.009, 15102499

[ref14] Frumkin H. Bratman G. N. Breslow S. J. Cochran B. Kahn P. H. Lawler J. J. . (2017). Nature contact and human health: a research agenda. Environ. Health Perspect. 125:075001. doi: 10.1289/EHP1663 PMC5744722 28796634

[ref15] Hartig T. Evans G. W. Jamner L. D. Davis D. S. Garling T. (2003). Tracking restoration in natural and urban field settings. J. Environ. Psychol. 23, 109–123. doi: 10.1016/S0272-4944(02)00109-3

[ref16] Hartig T. Korpela K. Evans G. W. Garling T. (1997). A measure of restorative quality in environments. Scand. Housing Plann. Res. 14, 175–194. doi: 10.1080/02815739708730435

[ref17] Hartig T. Mitchell R. de Vries S. Frumkin H. (2014). Nature and health. Annu. Rev. Public Health 35, 207–228. doi: 10.1146/annurev-publhealth-032013-182443, 24387090

[ref18] Herzog T. R. Maguire C. P. Nebel M. B. (2003). Assessing the restorative components of environments. J. Environ. Psychol. 23, 159–170. doi: 10.1016/S0272-4944(02)00113-5

[ref19] Holleman G. A. Hooge I. T. C. Kemner C. Hessels R. S. (2020). The “real-world approach” and its problems: a critique of the term ecological validity. Front. Psychol. 11:721. doi: 10.3389/fpsyg.2020.00721, 32425850 PMC7204431

[ref20] Jasper H. H. (1958). The ten-twenty electrode system of the international federation. Electroencephalogr. Clin. Neurophysiol. 10, 371–375. 10590970

[ref21] Jiang B. Chang C. Y. Sullivan W. C. (2014). A dose of nature: tree cover, stress reduction, and gender differences. Landsc. Urban Plan. 132, 26–36. doi: 10.1016/j.landurbplan.2014.08.005

[ref22] Joye Y. van den Berg A. E. (2011). Is love for green in our genes? A critical analysis of evolutionary assumptions in restorative environments research. Urban For. Urban Green. 10, 261–268. doi: 10.1016/j.ufug.2011.07.004

[ref23] Kaplan S. (1995). The restorative benefits of nature: toward an integrative framework. J. Environ. Psychol. 15, 169–182. doi: 10.1016/0272-4944(95)90001-2

[ref24] Kaplan R. Kaplan S. (1989). The Experience of Nature: A Psychological Perspective. Cambridge: Cambridge University Press.

[ref25] Klimesch W. (1999). EEG alpha and theta oscillations reflect cognitive and memory performance: a review and analysis. Brain Res. Rev. 29, 169–195. doi: 10.1016/S0165-0173(98)00056-3, 10209231

[ref26] Korpela K. M. Hartig T. Kaiser F. G. Fuhrer U. (2001). Restorative experience and self-regulation in favorite places. Environ. Behav. 33, 572–589. doi: 10.1177/00139160121973133

[ref27] Latifzadeh K. Emami P. Emami F. Duraisamy S. Leiva L. A. (2026) Pixels, plants, and people: affective evaluation of urban green spaces Proceedings of the 2026 CHI Conference on Human Factors in Computing Systems New York, NY Association for Computing Machinery

[ref28] Laumann K. Garling T. Stormark K. M. (2001). Rating scale measures of restorative components of environments. J. Environ. Psychol. 21, 31–44. doi: 10.1006/jevp.2000.0179

[ref29] Li D. Sullivan W. C. (2016). Impact of views to school landscapes on recovery from stress and mental fatigue. Landsc. Urban Plan. 148, 149–158. doi: 10.1016/j.landurbplan.2015.12.015

[ref30] Li Y. Yan X. (2026). Research on the emotional generation mechanism and optimization pathways of traditional village landscapes in northeastern Hubei based on EEG measurements. Front. Psychol. 17:1755492. doi: 10.3389/fpsyg.2026.1755492, 41798008 PMC12961987

[ref31] Lindal P. J. Hartig T. (2015). Effects of urban street vegetation on judgments of restoration likelihood. Urban For. Urban Green. 14, 200–209. doi: 10.1016/j.ufug.2015.02.001

[ref32] Markevych I. Schoierer J. Hartig T. Chudnovsky A. Hystad P. Dzhambov A. M. . (2017). Exploring pathways linking greenspace to health: theoretical and methodological guidance. Environ. Res. 158, 301–317. doi: 10.1016/j.envres.2017.06.028, 28672128

[ref33] McNair D. M. Lorr M. Droppleman L. F. (1971). Manual for the Profile of Mood States. San Diego, CA: Educational and Industrial Testing Service.

[ref34] Menser T. Baek J. Siahaan J. Kolman J. M. Delgado D. Kash B. (2021). Validating visual stimuli of nature images and identifying the representative characteristics. Front. Psychol. 12:685815. doi: 10.3389/fpsyg.2021.685815, 34566764 PMC8460908

[ref35] Ode A. Tveit M. S. Fry G. (2008). Capturing landscape visual character using indicators: touching base with landscape aesthetic theory. Landsc. Res. 33, 89–117. doi: 10.1080/01426390701773854

[ref36] Parsons R. Daniel T. C. (2002). Good looking: in defense of scenic landscape aesthetics. Landsc. Urban Plan. 60, 43–56. doi: 10.1016/S0169-2046(02)00051-8

[ref37] Ray W. J. Cole H. W. (1985). EEG alpha activity reflects attentional demands, and beta activity reflects emotional and cognitive processes. Science 228, 750–752. doi: 10.1126/science.3992243, 3992243

[ref38] Rhee J. H. Schermer B. Han G. Park S. Y. Lee K. H. (2023). Effects of nature on restorative and cognitive benefits in indoor environment. Sci. Rep. 13:13199. doi: 10.1038/s41598-023-40408-x, 37580348 PMC10425438

[ref39] Ribeiro I. J. S. Pereira R. Freire I. V. de Oliveira B. G. Casotti C. A. Boery E. N. (2018). Stress and quality of life among university students: a systematic literature review. Health Prof. Educ. 4, 70–77. doi: 10.1016/j.hpe.2017.03.002

[ref40] Rombach R. Blattmann A. Lorenz D. Esser P. Ommer B. (2022) High-resolution image synthesis with latent diffusion models Proceedings of the IEEE/CVF Conference on Computer Vision and Pattern Recognition 10684–10695

[ref41] Song R. Chen Q. Zhang Y. Jia Q. A. He H. Gao T. . (2022). Psychophysiological restorative potential in cancer patients by virtual reality (VR)-based perception of natural environment. Front. Psychol. 13:1003497. doi: 10.3389/fpsyg.2022.1003497, 36300069 PMC9589456

[ref42] Spano G. Theodorou A. Reese G. Carrus G. Sanesi G. Panno A. (2023). Virtual nature, psychological and psychophysiological outcomes: a systematic review. J. Environ. Psychol. 89:102044. doi: 10.1016/j.jenvp.2023.102044

[ref43] Tang R. Zhao X. Guo Z. (2024). Place perception and restorative experience of recreationists in the natural environment of rural tourism. Front. Psychol. 15:1341956. doi: 10.3389/fpsyg.2024.1341956, 38845763 PMC11153826

[ref44] Tveit M. Ode A. Fry G. (2006). Key concepts in a framework for analysing visual landscape character. Landsc. Res. 31, 229–255. doi: 10.1080/01426390600783269

[ref45] Twohig-Bennett C. Jones A. (2018). The health benefits of the great outdoors: a systematic review and meta-analysis of greenspace exposure and health outcomes. Environ. Res. 166, 628–637. doi: 10.1016/j.envres.2018.06.030, 29982151 PMC6562165

[ref46] Ulrich R. S. (1984). View through a window may influence recovery from surgery. Science 224, 420–421. doi: 10.1126/science.6143402, 6143402

[ref47] Ulrich R. S. Simons R. F. Losito B. D. Fiorito E. Miles M. A. Zelson M. (1991). Stress recovery during exposure to natural and urban environments. J. Environ. Psychol. 11, 201–230. doi: 10.1016/S0272-4944(05)80184-7

[ref48] van den Berg A. E. Koole S. L. van der Wulp N. Y. (2003). Environmental preference and restoration: (how) are they related? J. Environ. Psychol. 23, 135–146. doi: 10.1016/S0272-4944(02)00111-1

[ref49] Velarde M. D. Fry G. Tveit M. (2007). Health effects of viewing landscapes: landscape types in environmental psychology. Urban For. Urban Green. 6, 199–212. doi: 10.1016/j.ufug.2007.07.001

[ref50] White M. P. Alcock I. Grellier J. Wheeler B. W. Hartig T. Warber S. L. . (2019). Spending at least 120 minutes a week in nature is associated with good health and wellbeing. Sci. Rep. 9:7730. doi: 10.1038/s41598-019-44097-3, 31197192 PMC6565732

