# Correction: Foreground, midground, and restoration: EEG and preference responses to depth-controlled rural scenes

**DOI:** 10.3389/fpsyg.2026.1989995

**Published:** 2026-09-18

**Authors:** Xing Xiong, Shanrui Yang, Xinyi Jiang, Qinghai Zhang

**Affiliations:** Department of Landscape Architecture, Nanjing Agricultural University, Nanjing, China

**Keywords:** depth estimation, EEG, flux, landscape preference, psychological restoration, rural landscape, stress recovery

The funder Ministry of Education Humanities and Social Sciences Youth Foundation Project, Grant No. 24YJC760143 was erroneously omitted. The correct **Funding** statement reads:

The author(s) declared that financial support was received for this work and/or its publication. This research was supported by the Ministry of Education Humanities and Social Sciences Youth Foundation Project: “Mechanisms and Planning Design Strategies for Slow-Mobility Environments in Promoting Public Health Under the ‘City-Park Integration' Framework” (Grant No. 24YJC760143) and the Ministry of Education of China's Fund for Humanities and Social Sciences Research (Grant No. 23YJAZH200).

The original version of this article has been updated.

