# Supplementary material for: Foreground, midground, and restoration: EEG and preference responses to depth-controlled rural scenes
Source: Front Psychol. 2026 Jul 27;17:1891123. doi: 10.3389/fpsyg.2026.1891123 (PMC13453773; doi:10.3389/fpsyg.2026.1891123)
Supplement: Supplementary file 1 [file Table_1.pdf]

Supplementary

Table 1. Full list of the 27 final AI-generated rural landscape stimuli.

Stimulus images	Edited layer(s)	Valid. index	Depth clarity	Matched contrast
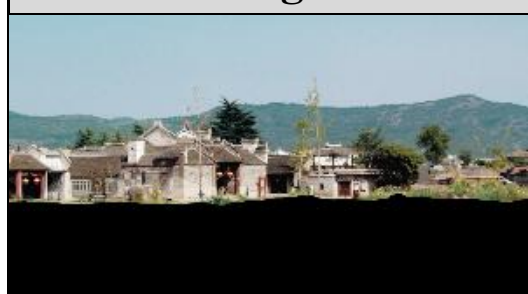	None (base only)	4.22	4.19	No
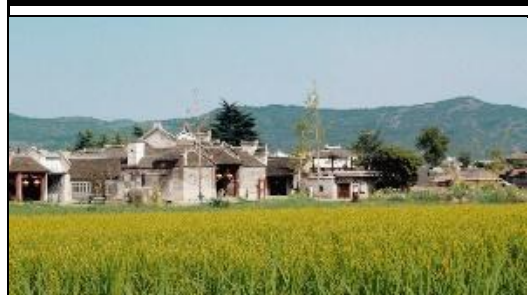	Midground only	4.32	4.09	No
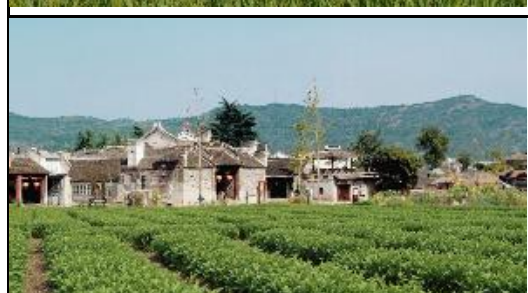	Midground only	4.15	4.06	No
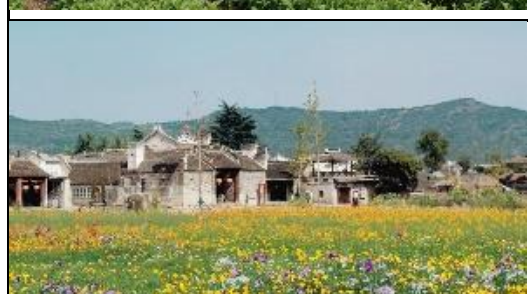	Midground only	4.18	3.94	No
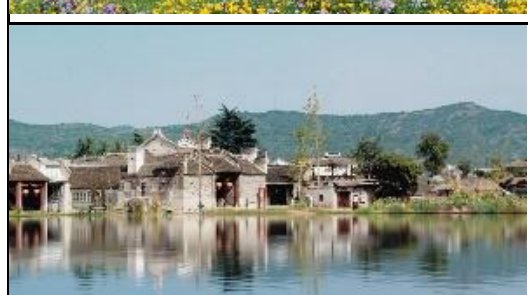	Foreground only	3.94	3.66	No
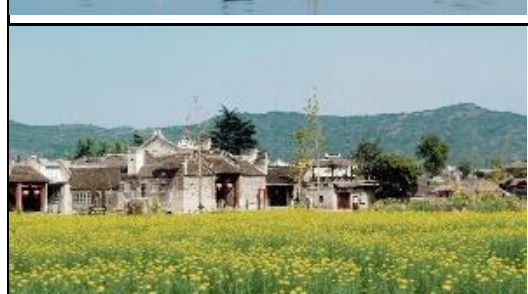	Midground only	4.15	3.82	No
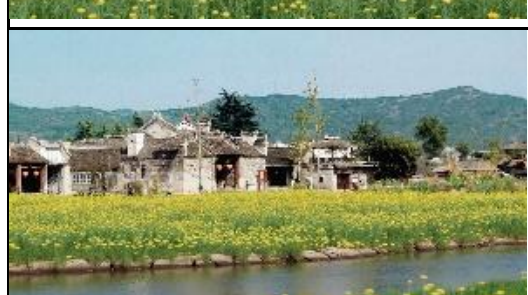	Fg + Mg	4.20	3.91	Yes
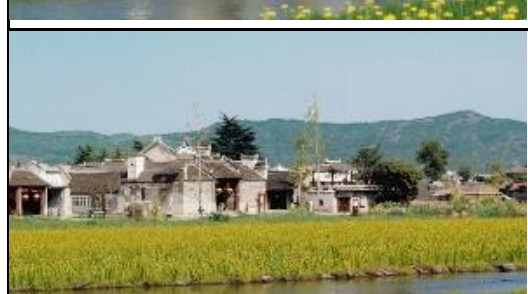	Fg + Mg	4.26	4.15	Yes
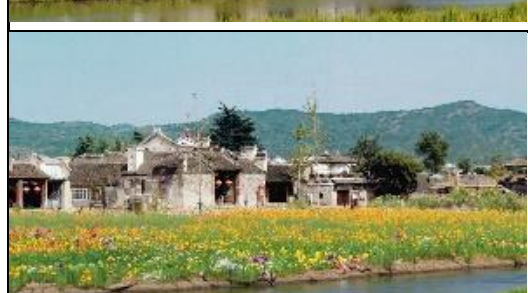	Fg + Mg	3.91	3.70	Yes
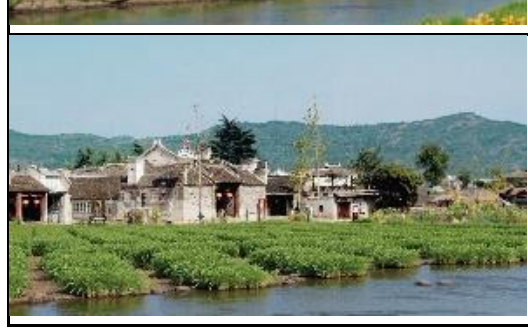	Fg + Mg	4.26	4.17	Yes

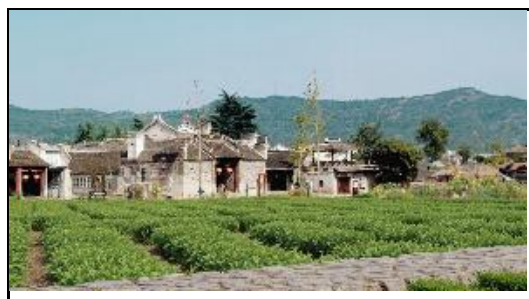	Fg + Mg	3.88	3.67	Yes
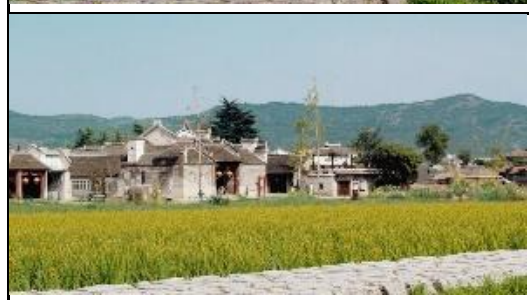	Fg + Mg	4.04	3.81	Yes
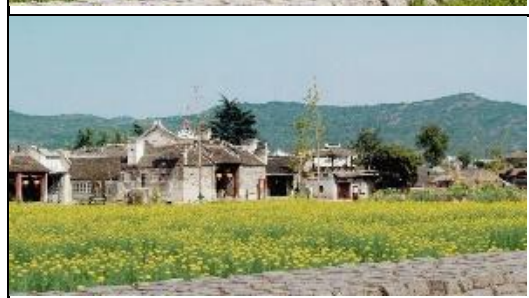	Fg + Mg	3.96	3.82	Yes
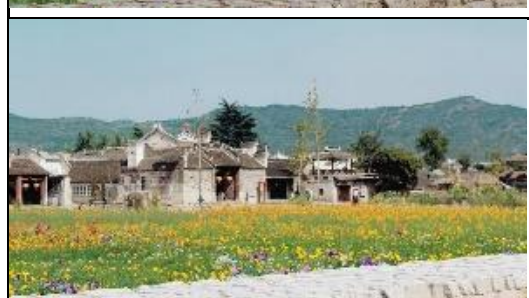	Fg + Mg	4.08	4.10	Yes
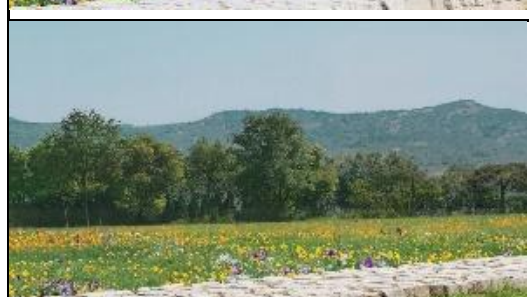	Fg + Mg	3.92	3.84	Yes
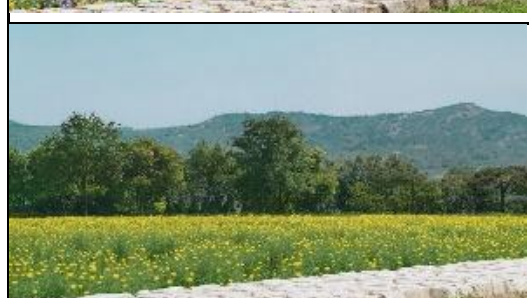	Fg + Mg	3.98	3.66	Yes
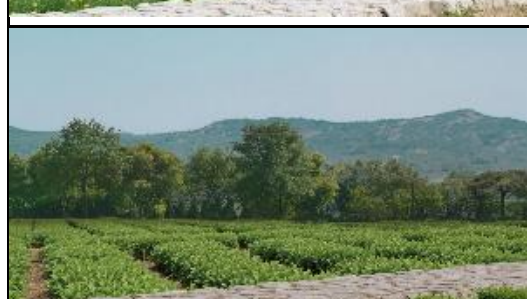	Fg + Mg	3.99	3.80	Yes
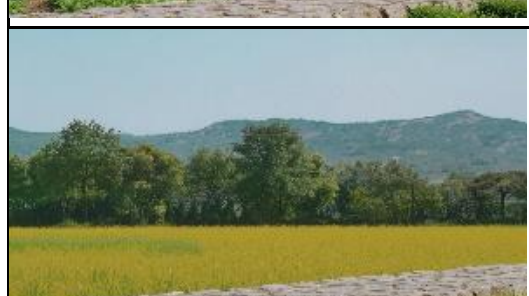	Fg + Mg	4.03	3.88	Yes
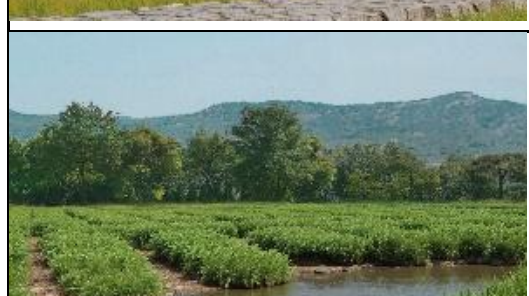	Fg + Mg	4.04	3.85	Yes
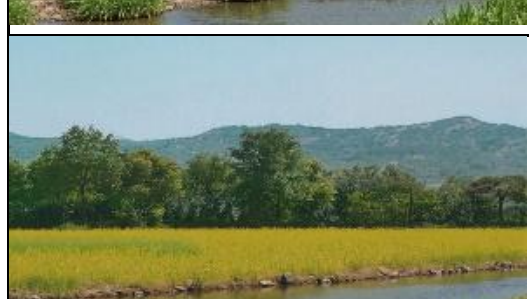	Fg + Mg	3.97	3.79	Yes
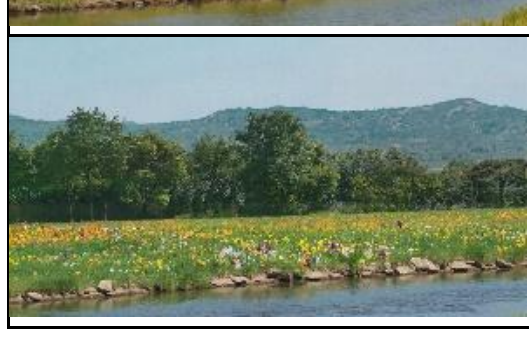	Fg + Mg	3.88	3.74	Yes

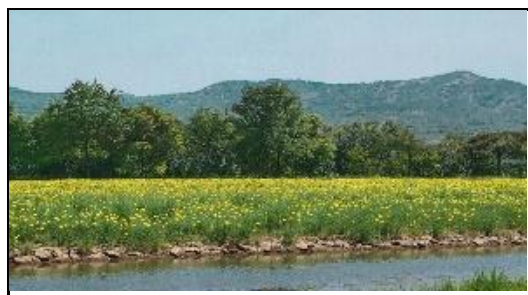	Fg + Mg	3.97	3.74	Yes
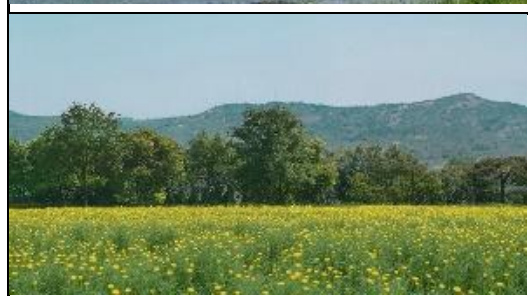	Midground only	4.13	3.87	No
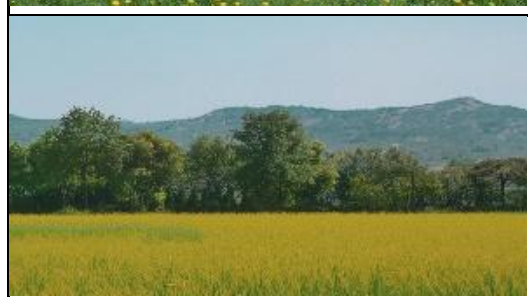	Midground only	4.04	3.73	No
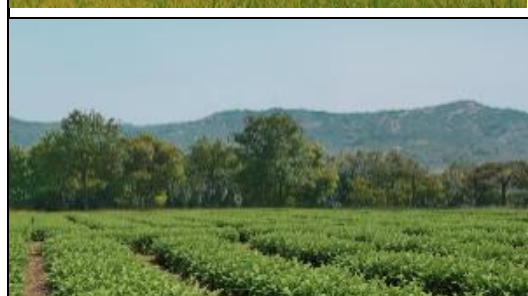	Midground only	4.18	4.07	No
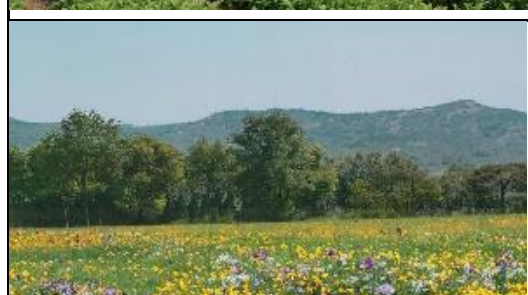	Midground only	4.16	4.07	No
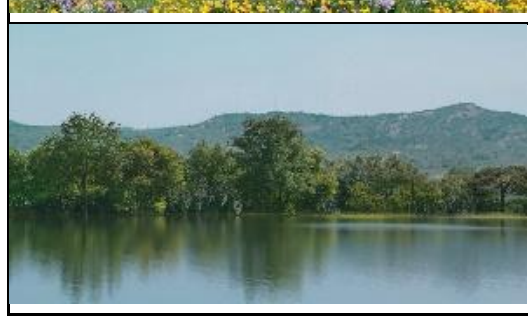	Foreground only	3.99	4.04	No
