# Supplementary material for: Foreground, midground, and restoration: EEG and preference responses to depth-controlled rural scenes
Source: Front Psychol. 2026 Jul 27;17:1891123. doi: 10.3389/fpsyg.2026.1891123 (PMC13453773; doi:10.3389/fpsyg.2026.1891123)
Supplement: Supplementary file 2 [file Table_2.pdf]

Supplementary

Table 2. EEG acquisition, preprocessing, and analytical parameters.

Parameter	Description
EEG system	32-channel eego™ mylab portable EEG system, model ES-303, ANT Neuro, Enschede, Netherlands
Cap and electrodes	waveguard™ cap with Ag/AgCl sintered electrodes
Montage	Extended international 10–20 system covering frontal, frontocentral, central, centroparietal, temporal, parietal, parieto-occipital, and occipital scalp regions
Electrode positions	Fp1, Fp2, F7, F3, Fz, F4, F8, FC5, FC1, FC2, FC6, C3, Cz, C4, CP5, CP1, CP2, CP6, T7, T8, P7, P3, Pz, P4, P8, POz, O1, Oz, O2
Ground and reference	AFz ground; CPz online reference
Recording settings	Data were preprocessed using EEGLAB, including electrode-coordinate checking, filtering, artifact rejection, ICA-based artifact removal, epoching, and spectral analysis
Filtering	0.3–30 Hz band-pass filter and 50 Hz notch filter
Epoch definition	Each image was segmented into a 10 s epoch; the first 1 s was excluded, and the remaining 9 s was analyzed
Frequency bands	α: 8–13 Hz; β: 14–30 Hz
